# Diagnosis of Bladder Diverticula with Point-of-Care Ultrasound

**DOI:** 10.5811/cpcem.2021.7.53199

**Published:** 2021-10-26

**Authors:** Shadi Lahham, Salvador Gutierrez

**Affiliations:** University of California, Irvine, Department of Emergency Medicine, Orange, California

**Keywords:** bladder diverticulum, laparoscopy

## Abstract

**Case Presentation:**

A 65-year-old male presented to the emergency department with symptoms including fever, abnormal urinalysis, and elevated post-void residual. Point-of-care ultrasound was used to rapidly diagnose a bladder diverticulum. The patient was subsequently seen by urology for outpatient bladder repair.

**Discussion:**

Bladder diverticula, an out-pouching of the bladder, may occur congenitally or as a result of various bladder conditions and/or surgery. Although bladder diverticula are rare and often asymptomatic, severe complications including frequent recurring urinary tract infections may arise depending on the patient.

## CASE PRESENTATION

A 65-year-old male with a history of benign prostatic hypertrophy presented to the emergency department with generalized weakness, low-grade fever, and urinary frequency. Point-of-care ultrasound (POCUS) demonstrated a post-void residual greater than 500 milliliters, measured with ultrasound machine calculation software, and a large abnormality of the urinary bladder (Image, Video). No hydronephrosis was seen on renal ultrasound. Chart review showed several prior visits for urinary tract infections (UTI) and one hospitalization for sepsis due to pyelonephritis. Given multiple previous infections and sensation of incomplete emptying, a POCUS was performed and aided in making the diagnosis. The patient was treated with intravenous antibiotics and discharged home. Ultimately, given the symptomatic nature of the bladder diverticula with multiple previous UTIs, urology repaired the defect as an outpatient procedure.

## DISCUSSION

A bladder diverticulum is an out-pouching of the bladder that occurs when a part of the bladder lining protrudes through a weakness in the bladder wall. These occur either congenitally or as an acquired condition from bladder outlet obstruction, neurogenic bladder conditions, or from prior bladder surgery.^1^ The prevalence of congenital diverticula is approximately 1.7%.^2^ The incidence increases with age and is most common in men with benign prostatic hypertrophy at a rate of up to 6%.^3^ The male to female ratio of 9:1 reflects this finding.^4^ Because bladder diverticula are typically asymptomatic, they are usually discovered on evaluations for UTIs, hematuria, or lower urethral tract symptoms.

Management of bladder diverticula depends on the complications that arise. Nonoperative, conservative management includes treatment with antibiotics for UTIs and avoidance of medications that cause urinary retention, such as opioids. Malignancy is a feared complication as the diverticula lacks a muscular wall outside the mucosal layer allowing metastatic spread more rapidly. Open or laparoscopic surgical correction options exist and are chosen based on several factors such as malignancy, size, and surgeon experience.^1^ This diagnosis can be made with POCUS and may explain the etiology of patients with recurrent UTIs. It is important to note that complete imaging of the bladder may be necessary to capture a definitive image of the break in the bladder wall.

**CPC-EM Capsule**

What do we already know about this clinical entity?

*Bladder diverticulum is an out-pouching of the bladder that may be acquired or congenital.*

What is the major impact of the image(s)?

*Bladder diverticula, which can be identified using point-of-care ultrasound, should be considered in patients with repeat urinary tract infections (UTI).*

How might this improve emergency medicine practice?

*Point-of-care ultrasound can help identify bladder diverticula in patients with repeat UTIs.*

## Supplementary Information

Video. A coronal, or long-axis, view of the bladder with an out-pouching bladder diverticulum on the right side of the screen.

## Figures and Tables

**Image:**
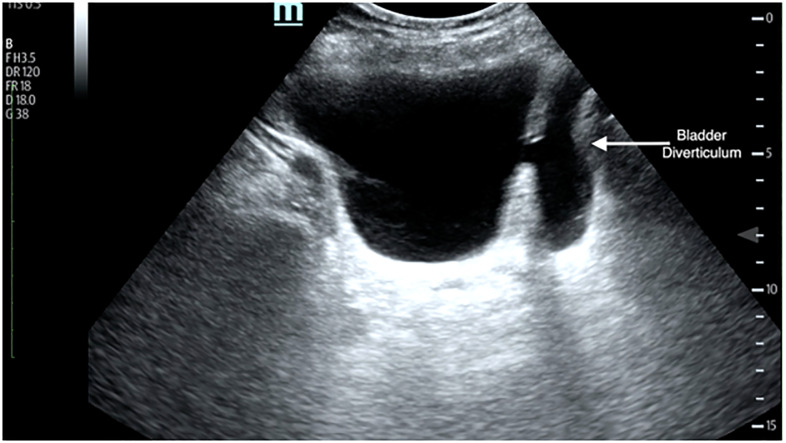
Ultrasound demonstrating a bladder diverticulum, with the out-pouching (arrow) to the right of the bladder.
